# Extracellular Vesicle Associated miRNAs Regulate Signaling Pathways Involved in COVID-19 Pneumonia and the Progression to Severe Acute Respiratory Corona Virus-2 Syndrome

**DOI:** 10.3389/fimmu.2021.784028

**Published:** 2021-12-09

**Authors:** Agnes S. Meidert, Stefanie Hermann, Florian Brandes, Benedikt Kirchner, Dominik Buschmann, Jean-Noël Billaud, Matthias Klein, Anja Lindemann, Elisa Aue, Gustav Schelling, Michael W. Pfaffl, Marlene Reithmair

**Affiliations:** ^1^ Department of Anesthesiology, University Hospital, LMU, Munich, Germany; ^2^ Division of Animal Physiology and Immunology, School of Life Sciences Weihenstephan, Technical University of Munich, Freising, Germany; ^3^ QIAGEN Digital Insights, Redwood City, CA, United States; ^4^ Department of Neurology, University Hospital, LMU, Munich, Germany; ^5^ Institute of Human Genetics, University Hospital, LMU, Munich, Germany

**Keywords:** COVID-19, community acquired pneumonia, Severe Acute Respiratory Corona Virus-2 Syndrome, extracellular vesicles, small RNA sequencing, cell-free microRNAs, sepsis, ARDS

## Abstract

**Background:**

Extracellular vesicles (EVs) are mediators of cell-to-cell communication in inflammatory lung diseases. They function as carriers for miRNAs which regulate mRNA transcripts and signaling pathways after uptake into recipient cells. We investigated whether miRNAs associated with circulating EVs regulate immunologic processes in COVID-19.

**Methods:**

We prospectively studied 20 symptomatic patients with COVID-19 pneumonia, 20 mechanically ventilated patients with severe COVID-19 (severe acute respiratory corona virus-2 syndrome, ARDS) and 20 healthy controls. EVs were isolated by precipitation, total RNA was extracted, profiled by small RNA sequencing and evaluated by differential gene expression analysis (DGE). Differentially regulated miRNAs between groups were bioinformatically analyzed, mRNA target transcripts identified and signaling networks constructed, thereby comparing COVID-19 pneumonia to the healthy state and pneumonia to severe COVID-19 ARDS.

**Results:**

DGE revealed 43 significantly and differentially expressed miRNAs (25 downregulated) in COVID-19 pneumonia when compared to controls, and 20 miRNAs (15 downregulated) in COVID-19 ARDS patients in comparison to those with COVID-19 pneumonia. Network analysis for comparison of COVID-19 pneumonia to healthy controls showed upregulated miR-3168 (log2FC=2.28, p_adjusted_<0.001), among others, targeting interleukin-6 (IL6) (25.1, 15.2 - 88.2 pg/ml in COVID-19 pneumonia) and OR52N2, an olfactory smell receptor in the nasal epithelium. In contrast, miR-3168 was significantly downregulated in COVID-19 ARDS (log2FC=-2.13, p_adjusted_=0.003) and targeted interleukin-8 (CXCL8) in a completely activated network. Toll-like receptor 4 (TLR4) was inhibited in COVID-19 pneumonia by miR-146a-5p and upregulated in ARDS by let-7e-5p.

**Conclusion:**

EV-derived miRNAs might have important regulative functions in the pathophysiology of COVID-19: CXCL8 regulates neutrophil recruitment into the lung causing epithelial damage whereas activated TLR4, to which SARS-CoV-2 spike protein binds strongly, increases cell surface ACE2 expression and destroys type II alveolar cells that secrete pulmonary surfactants; both resulting in pulmonary-capillary leakage and ARDS. These miRNAs may serve as biomarkers or as possible therapeutic targets.

## Introduction

The clinical course of COVID-19 appears to be highly variable and affects patients with differences in age and sex with a varying degree of severity (1, 2). Whereas most infected individuals are asymptomatic or develop only mild symptoms, other progress to interstitial pneumonia (3) requiring hospitalization and supplemental oxygen therapy (1). More severely affected patients show rapid disease progression and develop the *Severe Acute Respiratory Corona Virus-2 Syndrome* fulfilling criteria for the *Acute Respiratory Distress Syndrome* (ARDS) (4) resulting in pulmonary failure and multiple organ dysfunction (5). ARDS is a highly significant predictor for death in COVID-19 patients as ARDS is seen in more than 90% of non-survivors (6).

Although there are many clinical similarities between COVID-19 and community acquired pneumonia and non-SARS-CoV-2 associated ARDS, there are important discrepancies in the time course and aggressivity of these diseases. In COVID-19 patients, ARDS appears to be more severe than seen in other critically ill patients. One out of 4 patients with COVID-19 pneumonia develops this form of severe lung injury (6), a much higher ratio than in patients with community acquired pneumonia or sepsis of bacterial origin. Despite comparable manifestations of these disorders, there must be crucial differences in signaling pathways in SARS-CoV-2 infections as compared to other pathogens that also result in pulmonary injury, sepsis and multiple organ failure.

Recent research indicates that non-coding RNAs co-precipitating with circulating extracellular vesicles affect many disorders including infectious pulmonary diseases (7–10). Non-coding RNAs include an approximately 22-nt-long subclass of RNAs designated as microRNAs (miRNAs) that modulate posttranscriptional gene expression. Viral infections often alter cellular miRNA expression profiles and RNA viruses are known to interact directly with cellular miRNAs to increase virus replication (11). Extracellular vesicles, released by almost all cell types, are important carriers of many molecules including proteins and non-coding RNAs, in particular miRNAs, in the blood stream and body fluids (12). Carried by vesicles, signaling molecules (e.g. miRNAs) can either bind to neighboring cells to change the local microenvironment (13) or travel with the blood stream to distant target cells, followed by cellular uptake (14) which results in changes in gene expression and the physiology in the recipient cells.

The pathogenesis of COVID-19 is not limited to toxic viral effects on tissues and blood cells but also involves the host’s inflammatory response and immunomodulation by the virus itself. Consequently, we assumed that the analysis of expression patterns of host-miRNAs derived from circulating extracellular vesicles could help to delineate specific signaling pathways in COVID-19 different from other forms of pulmonary injury resulting from bacterial community acquired pneumonia or sepsis. We therefore examined host-miRNAs derived from circulating extracellular vesicles from patients with COVID-19 pneumonia, COVID-19 ARDS, community acquired pneumonia of bacterial origin and ARDS due to bacterial sepsis. With bioinformatically constructed canonical pathways comparing miRNA expression patterns between these disorders, we aimed to characterize differences in molecular signaling between COVID-19 and other infectious pulmonary disorders with a comparable clinical phenotype. This approach aimed to detect novel and more specific biomarkers for COVID-19 or even alternative therapeutic approaches targeting the identified specific COVID-19 regulated pathways.

## Methods

### Patients and Controls

We studied 100 individuals in total. Of those, 20 symptomatic patients had PCR-confirmed COVID-19 pneumonia and 20 had developed COVID-19 ARDS requiring ICU therapy and mechanical ventilation. Twenty healthy, non-vaccinated individuals without a history of COVID-19 were included as controls. As an additional comparison group to the COVID-19 patients, we included small RNA sequencing data from 12 patients hospitalized with bacterial community acquired pneumonia and 28 ICU patients with sepsis associated ARDS (23 had septic shock) from an earlier study (9).

Patients with COVID-19 pneumonia were prospectively recruited between 03/2020 and 04/2020 soon after admittance to the isolation facility of the University Hospital of Munich, had at least one positive nasal swap for SARS-CoV-19 virus (SARS-CoV-2-RNA PCR test, RdRP-Gen IP4) and typical symptoms. Patients with community acquired pneumonia were recruited between 04/2017 and 01/2019. None of these patients had evidence of a viral infection (RSV-RNA, Influenza Type-A/-B-RNA). RNA samples from these comparison groups were already available at study onset from the previous investigation (9). Sepsis in the earlier study was defined according to the Third International Task Force for Sepsis and Septic Shock (Sepsis-3) (15). Inclusion and exclusion criteria for the study subgroups are presented in **Table 1**.

**Table 1:** Study inclusion and exclusion criteria according to study groups.

Criteria	COVID-19 Pneumonia	COVID-19 ARDS	Community acquired pneumonia (bacterial origin)	Sepsis induced ARDS	Healthy controls
**Inclusion**	Positive nasal swap for SARS-CoV-19 virus (SARS-CoV-2-RNA PCR test, RdRP-Gen IP4) with typical symptoms for COVID-19 infection. CURB-65^†^ ≥1 and clinical symptoms like fever, cough and dyspnea.	Positive nasal swap for SARS-CoV-19 virus Radiologic evidence of pulmonary involvement by CT-scan Mechanical ventilation because of ARDS	community acquired pneumonia without recent hospitalization or association with other healthcare facilities such as nursing homes, dialysis centers, and outpatient clinics. CURB-65^†^ ≥1 and clinical symptoms like fever, cough and dyspnea.	Sepsis or septic shock according to Sepsis-3 criteria (16) ARDS according to Berlin criteria (4)	Charlson Comorbidity Index = 0 (17)
**Exclusion**	No consent given by individuals or next-of-kin				
	Age <18				
	Pregnancy				
	Preexisting chronic infectious disorders (e.g., endocarditis, HIV or hepatitis)				
	Current tumor or malignant disorders				
	Limited patient’s life expectancy <6 months (independent of e.g., sepsis/SIRS, pneumonia or localized infection)				
	Immunosuppression or steroid therapy (e.g., autoimmune disease, transplantation)				

^†^Confusion, Urea, Respiratory Rate, Blood Pressure and Age (CURB-65) score for pneumonia severity (18).

We selected 5 comparisons of clinical interest: 1) healthy controls (baseline) compared to patients with COVID-19 pneumonia; 2) patients with COVID-19 pneumonia (baseline) compared to patients with COVID-19 ARDS; 3) patients with COVID-19 ARDS at admission to the ICU (baseline) compared to day 14 of ICU treatment; 4) patients with bacterial community acquired pneumonia (baseline) compared to patients with COVID-19 pneumonia; 5) patients with sepsis on the day of ICU-admission (baseline) compared to patients with COVID-19 ARDS.

### Statistical Analysis of Demographics and Clinical Data

No data on the effects of a SARS-CoV-2 infection on miRNA expression patterns in extracellular vesicles were available, therefore, the estimation of required case numbers to achieve significant results after correction for multiple comparison as part of the statistical plan was based on a previous study by our group which compared patients with bacterial community acquired pneumonia to healthy individuals (9). In this study with a group size of n=12 patients with pneumonia, the analysis of differential gene expression analysis (DGE) data resulted in 29 significantly regulated miRNAs between the two groups. As the impact of a SARS-CoV-2 infection in comparison to a bacterial pulmonary infection on miRNA expression levels was not known, we increased the planned sample size for the COVID-19 pneumonia group to 20 and recruited an equal number of individuals for the other groups.

Demographic and clinical data between groups were compared using the non-parametric Mann-Whitney U test. The Chi-square or Fisher’s exact test was used for comparison of categorical variables.

Data analysis was performed using Python version 3.7 (Python Software Foundation, Beaverton, USA) and SPSS (IBM SPSS Statistics for Windows, version 25; IBM Corp., Armonk, N.Y., USA). Data in the text and in tables are reported as median and interquartile range (IQR). All statistical tests were two-tailed and a p-value < 0.05 was considered statistically significant.

### Blood Sampling and Processing

Blood from patients with COVID-19 pneumonia, community acquired pneumonia and from volunteers was drawn by venipuncture (21-gauge needles) or aspiration through venous catheters (Safety-Multifly, Sarstedt AG & Co, Nümbrecht, Germany). In ICU patients (COVID-19 ARDS or sepsis), blood sampling was performed at admission to the intensive care facility using 20-gauge catheters within the radial artery (8 cm polyethylene catheter, Vygon, Aachen, Germany). In a previous study, we showed that arterial vs. venous blood sampling has non-significant effects on EV miRNA expression (19). Blood was drawn into 9 ml serum collection tubes (S-Monovette, Sarstedt AG&Co, Nümbrecht, Germany), allowed to clot for 30 min and centrifuged at 3,400 x g for 10 min at room temperature. Within 10 min of separation, serum was aliquoted and immediately stored at -80°C. Whole blood samples for cellular RNA extraction were collected in PAXgene tubes (PAXgene, Qiagen, Hilden, Germany) in accordance with the supplier’s protocol.

### Isolation and Characterization of Precipitated Extracellular Vesicle Samples

Extracellular vesicles were precipitated from 1ml serum using an isolation kit (miRCURY Exosome Isolation Kit, Qiagen, Venlo, Netherlands) as previously described (19) followed by extraction of cell-free RNA. For qPCR, an EV isolation and RNA extraction were performed from a second 1 ml serum aliquot. Particle concentration and size distribution were evaluated by Nanoparticle Tracking Analysis on a ZetaView PMX 110 (Particle Metrix, Meerbusch, Germany) and visualized by transmission electron microscopy. Following the Shapiro-Wilk normality test, statistical significance of particle tracking analysis data were evaluated by ordinary one-way ANOVA followed by Tukey’s multiple comparison test using Graphpad Prism (version 8.3.1) with a significance level of p ≤ 0.05. Particle concentration and size were reported as mean values ± standard deviation. Additionally, precipitated vesicles were visualized after negative staining with 4% uranyl acetate by transmission electron microscopy using a Zeiss EM900 (Carl Zeiss Microscopy GmbH, Jena, Germany) with a wide-angle dual speed 2KCCD camera at 80 kV.

### RNA Processing and Sequencing

#### Small RNA Sequencing

For small RNA sequencing (RNAseq), libraries were prepared using the NEBNext Multiplex Small RNA Library Prep Set for Illumina (New England Biolabs Inc., Ipswich, USA) (20). Three ng of each cDNA library, as well as total libraries for samples with lower concentrations were pooled. Samples with < 3ng of cDNA were distributed equally over the different subgroups. Small RNAseq was performed in 50 cycles and single-end sequencing mode on the HiSeq2500 (Illumina Inc., San Diego, USA).

Quality control of small RNAseq data, trimming of adaptor sequences and alignment of reads was performed as described previously (19). Samples with a minimum of 5.10E+04 miRNA precursor reads were included for analyses. DGE analysis was conducted by DESeq2 (version 1.28.1) (21) for R (version 4.0.2) with the implemented normalization strategy based on library size correction and the Benjamini-Hochberg method to correct for the false discovery rate (FDR). miRNAs were filtered by setting a mean expression across all samples of ≥ 50 reads (baseMean), a minimum twofold up- or down-regulation (log2 fold change, log2FC ≥ 1 or log2FC ≤ -1) and adjusted p-value p_adjusted_ ≤ 0.05. Unsupervised clustering was performed by principal component analysis (PCA). Following Shapiro-Wilk normality test, statistical significance of library sizes was compared by parametric ordinary one-way ANOVA followed by Tukey’s multiple comparison, while miRNA mapping was evaluated with the non-parametric Kruskal-Wallis test followed by Dunn’s multiple comparison test. Both analyses were performed using Graphpad Prism (version 8.3.1) and p ≤ 0.05 was considered significant. Data were reported as mean values ± standard deviation.

#### Real Time Quantitative PCR

The most stably expressed miRNAs among all groups were evaluated from the small RNAseq data set as potential reference miRNAs by NormFinder (22). The following reference genes were identified: miR-30d-5p, miR-30e-5p, let-7i-5p, miR-148b-3p, 146b-5p, miR-425-5p, miR-24-3p and 125a-5p. Validations of the selected miRNAs were performed by RT-qPCR applying the LNA-optimized miRNA PCR system (miRCURY LNA RT kit, miRCURY LNA SYBR Green PCR kit, Qiagen, Venlo, The Netherlands). For reverse transcription, 4 µl of EV-associated total RNA was used as template for cDNA synthesis. qPCRs in a 10 µl reaction volume were prepared according to the manufacturer’s recommendation with the appropriate miRCURY LNA miRNA PCR Assays (Qiagen, Venlo, The Netherlands) for the candidate and reference miRNAs. The UniSp6 assay (Qiagen, Venlo, The Netherlands) was used as control for cDNA synthesis and PCR amplification. qPCR reactions were run on a Rotor-Gene Q thermal cycler (Qiagen, Hilden, Germany). qPCR miRNA results were normalized with the geometric mean of reference miRNAs. Representative mRNA targets (predicted by IPA analysis) were chosen for mRNA qPCR validation. For mRNA qPCR 750 ng of PAXgene extracted RNA was reverse transcribed using the QuantiTect reverse transcription kit (Qiagen, Hilden, Germany). qPCRs were carried out with the SsoAdvanced universal SYBR Supermix and PrimePCR assays (Biorad, Munich, Germany) in a 20 µl reaction volume with 10 ng template cDNA on a MiniOpticon device (Biorad, Munich, Germany). Reference gene candidate selection for mRNA qPCR was based on geNorm and NormFinder prediction using total RNA sequencing data of this cohort. For the comparison COVID-19 ARDS to COVID-19 pneumonia qPCR mRNA results were normalized with the geometric mean of reference genes HIF1A, ZFP36L1, ATP6V1B2, LAPTM5, NCF2 and RASSF2. For the comparison COVID-19 pneumonia to healthy controls qPCR mRNA results were normalized with ZFP36L1, LAPTM5 and NCF2. Reference genes were considered to have a p-value > 0.05 in Student’s t-test based on qPCR data. Mean Cq values of miRNA and mRNA qPCR were quantified relatively with the ΔΔCq method (23).

### Bioinformatic Characterization of Differentially Regulated Pathways Between Community Acquired Pneumonia, SARS-CoV-2 Associated ARDS and Sepsis

Ingenuity Pathway Analysis (IPA, version 57662101, autumn release 2020, Qiagen Digital Insights, Redwood, USA) was used for *in silico* identification of mRNA targets and causal networks based on the small RNAseq miRNA expression data. Possible gene targets and regulatory effects were identified using the IPA “*microRNA Target Filter”.* In a first approach, we compared RNAseq data from healthy controls to patients with COVID-19 pneumonia and COVID-19 pneumonia to COVID-19 associated ARDS. For this step, we filtered for miRNAs with high expression values (log2FC <-1.8 and >1.8) to identify maximally regulated miRNAs between these groups and constructed the respective signaling pathways. Experimentally confirmed or highly predicted relationships were used in this analysis. We then analyzed these patient groups focusing on *antimicrobial response* and *lung as a target organ* including only experimentally confirmed findings from the IPA data base to characterize more lung specific signaling networks. When limiting the filtering to experimentally confirmed findings, the software identifies only experimentally validated interactions between miRNAs and their potential mRNA targets from TarBase and miRecords and miRNA-mRNA related findings from the *Ingenuity Knowledge Base* which includes millions of manually curated contextual details and links to the original publications. In contrast, when selecting *predicted interactions* (further categorized into high and low prediction), miRNA–mRNA interactions from TargetScan are used in addition.

This was followed by constructing a network comparing miRNA expression values between day 0 (ICU admission) and day 14 in patients with COVID-19 ARDS to identify molecular signaling reflecting the partial recovery of patients from the viral infection using the abovementioned filter criteria. The next steps characterized molecular signaling networks in community acquired pneumonia of bacterial origin in comparison to COVID-19 pneumonia and between sepsis induced ARDS and COVID-19 ARDS.

### Ethical Approval

Ethics approval for the study was obtained from the Ethics Committee of the Medical Faculty of the Ludwig-Maximilians-University of Munich under protocols #551-14 and 20-299. All samples were pseudonymized during analyses. The study was conducted in accordance with approved guidelines and written informed consent to participate was obtained from each participant or the patient’s legal representative.

## Results

### Study Population

**Figure 1:**
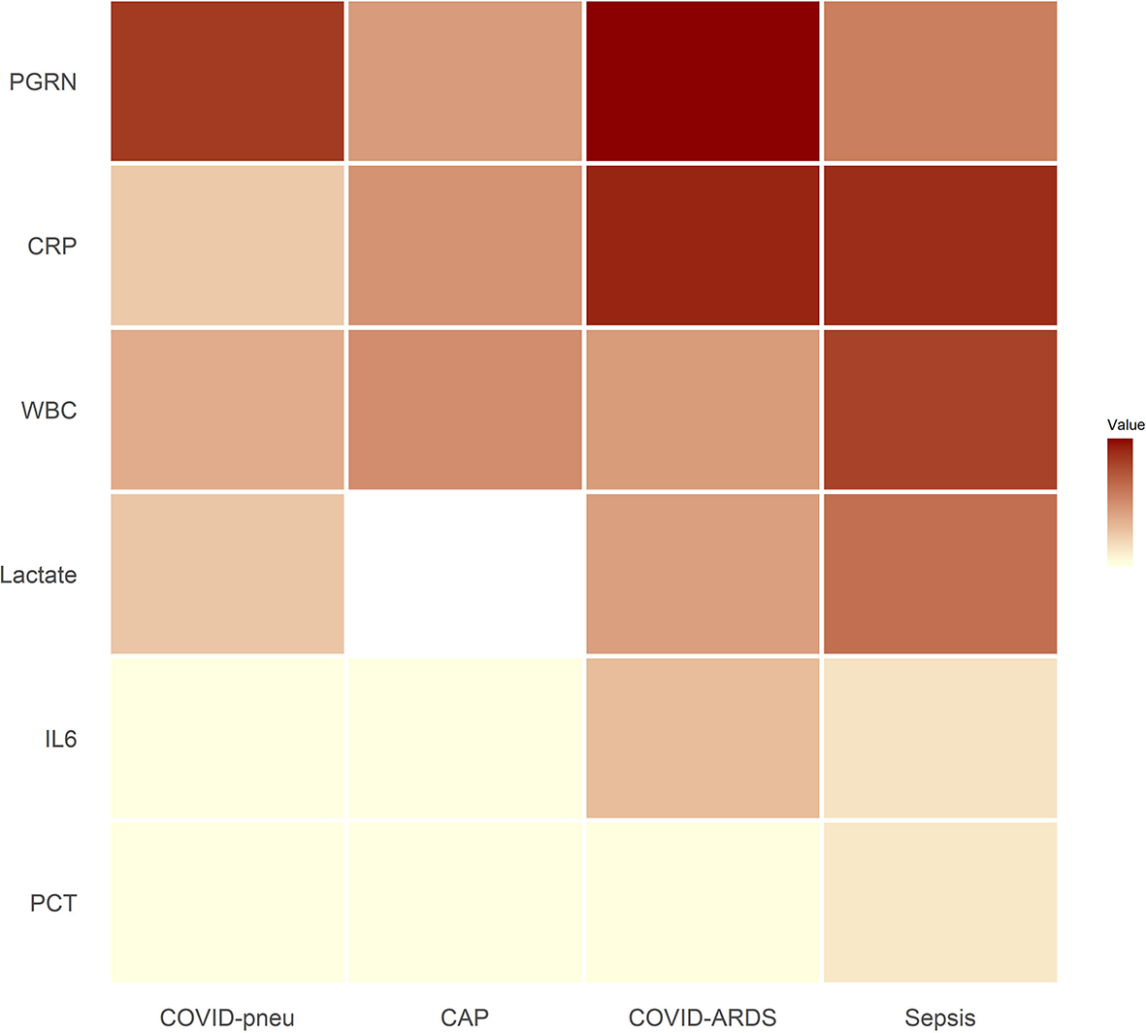
Heat map of inflammatory parameters of patient groups (medians). Outliers were set to the maximum of the remaining values. For community acquired pneumonia, no serum lactate was available. All associated numerical values and statistical comparisons of laboratory results between groups are presented in **Table 3**. PGRN, progranulin; CRP, C-reactive protein; WBC, leukocyte count; Lactate, serum lactate; IL6, interleukin-6; PCT, procalcitonin; COVID-pneu, COVID-19 pneumonia; CAP, community acquired pneumonia of bacterial origin; COVID-ARDS, COVID-19 acute respiratory distress syndrome; Sepsis, sepsis-related acute respiratory distress syndrome.

Small RNAseq was successful in 18 healthy controls, 15 patients with COVID-19 pneumonia and COVID-19 ARDS at ICU-admission, respectively and in 13 patients with COVID-19 ARDS on day 14 of ICU treatment. COVID-19 pneumonia and ARDS patients had comparable age, height and weight (**Table 2**). Patients with COVID-19 pneumonia and non-COVID-19 community acquired pneumonia had comparable median CURB-65 pneumonia severity scores at hospital admission (1, IQR 0-1.5 vs. 1, IQR 1-2), respectively, p=0.095. COVID-19 pneumonia patients had higher body mass index (BMI) values (26.1, IQR 24.5-30.7) than healthy controls (23.6, IQR 22.3-25.3, p=0.013) and healthy individuals were significantly younger than patients with COVID-19 pneumonia (35, 31-51.5 years *vs.* 63, 53-74 years, p<0.001). Patients with COVID-19 ARDS had a higher BMI than patients with sepsis. Furthermore, procalcitonin (an established biomarker for sepsis) and progranulin (24) were significantly different, whereas interleukin-6 and C-reactive-protein were comparable between these two groups (see **Figure 1** and **Table 3**).

**Table 2:** Characteristics of patients with successful next-generation-sequencing.

Parameter	COVID-19 pneumonia	Community acquired pneumonia	COVID-19 ARDS	Sepsis ARDS	Controls
Sex, n (male/female)	13/2	8/4	13/2	17/11	11/7
Pathogen (none detected/bacterial/viral)	0/0/15	10/2/0^*^	0/0/15	8/20/0^+^	–
Localisation (Abdominal/Other/Pulmonary)	0/0/15	0/0/13	0/0/15	11/2/15^+^	–
Death, n (%)	2 (13)	1 (8)	2 (13)	6 (21)	–
AKI, n (%)	0 (0)	0 (0)	9 (60)	15 (54)	–
Progression to ARDS, n (%)	7 (47)	0 (0)^*^			
					
Age (yrs)^1^	63 (53 - 74)	71 (64 - 85)^*^	65 (56.5 - 70.5)	66.5 (60.2 - 75)	35 (31 - 51.5)
Height (cm)^1^	178 (175 - 186)	170.5 (164.2 - 177.5)^*^	175 (171 - 180)	173 (165.5 - 177)	178.5 (170.5 – 184.3)
Weight (kg)^1^	86 (77.5 - 102)	81 (73.5 - 93.5)	85 (80 - 97.5)	75 (60 - 93.5)^+^	74 (65.3 – 86.8)
BMI (kg/m^2^)^1^	26.1 (24.5 - 30.7)	29.7 (24.4 - 30.6)	29.3 (25.9 - 34.1)	23.8 (21.8 - 28.9)^+^	23.6 (22.3 – 25.3)
Hospital stay (d)^1^	17 (12 - 31)	10 (7 - 14)^*^	35 (31 - 53)	29.5 (20 - 40.2)	–
Mechanical ventilation (d)^1^	–	–	18 (15.2 - 36.2)	10.5 (6.5 - 24.2)^+^	–
ICU stay (d)^1^			22.5 (18.2 - 42.8)	12 (9 - 32.5)^+^	–
Lowest paO2/FiO2 ratio at admission^1^			118 (89.9 - 147.5)	143 (109.2 - 188.5)^+^	–
Septic shock, n (%)			9 (60)	23 (82)	–
SOFA score^1^			11.0 (9.5 - 11.5)	11.5 (9 - 12.8)	–

AKI, acute kidney injury; ARDS, acute respiratory distress syndrome; BMI, Body Mass Index; SOFA, Sepsis-related organ failure assessment score. ^1^Data are median and quartiles.

^*^p < 0.05 compared to COVID-19 pneumonia patients; ^+^p<0.05 compared to COVID-19 ARDS patients.

**Table 3:** Laboratory results of different patient groups.

	COVID-19 pneumonia	Community acquired pneumonia	COVID-19 ARDS	Sepsis ARDS
Lactate (mmol/L)	1.1 (1.0 - 1.4)^3,4^		1.8 (1.6 - 2.0)	2.7 (1.6 - 4.0)
Procalcitonin (ng/ml)	0 (0 - 0.25)^3,4^	0.18 (0.14 - 0.62)^4^	0.90 (0.48 - 0.93)^4^	4.73 (2.22 - 9.40)
Leukocyte count (G/l)	6.68 (3.83 - 11.00)^4^	9.03 (6.85 - 12.50)	7.92 (7.35 - 12.55)	14.60 (8.21 - 18.53)
C-reactive protein (mg/dl)	6.2 (2.1 - 10.8)^3^	11.8 (2.4 - 19.1)^4^	22.7 (14.2 - 26.8)	22.0 (15.7 - 31.1)
Interleukin-6 (pg/ml)	25 (17 - 102) ^3,4^	12 (0 - 29) ^3,4^	492 (247 - 600)	236 (97 - 1762)
Serum Creatinine (mg/dl)	0.9 (0.8 - 1.0)^2,4^	1.2 (1.1 - 1.6)	1.1 (1.0 - 1.3)	1.4 (0.8 - 2.0)
Urea (mg/dl)	26 (23 - 30)^4^	40 (27 - 75) ^3^	19 (14 - 28)^4^	53 (38 - 80)
Progranulin (ng/ml)	71 (55 - 101)^2,4^	37 (34 - 41)^3,4^	85 (73 - 92)^4^	48 (36 - 72)

Data are median and interquartile range (P25-P75).

P < 0.05 compared to: ^2^Community acquired pneumonia; ^3^COVID-19 ARDS; ^4^Sepsis ARDS. Statistical tests used: Kruskal-Wallis test and Dunn’s test for multiple comparisons.

### Characteristics of Precipitated Extracellular Vesicles

**Figure 2:**
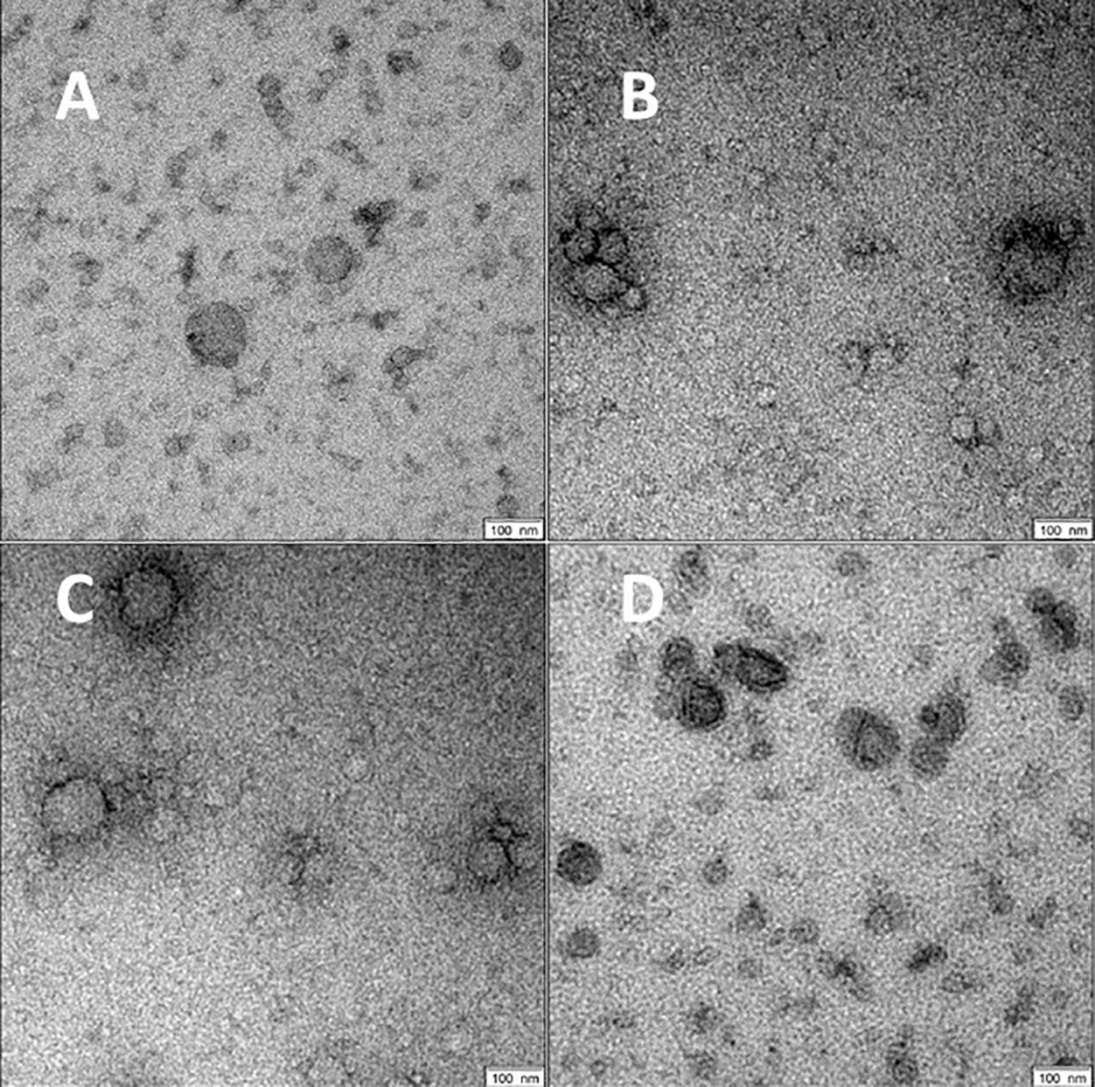
Morphology of serum EVs by transmission electron microscopy. Images are representative for at least two biological replicates for controls **(A)**, COVID-19 pneumonia **(B)**, COVID-19 ARDS at admission to the ICU **(C)** and at day 14 of ICU treatment **(D)**. Scale bars shown at the right lower corner of the images are 100 nm.

**Figure 3:**
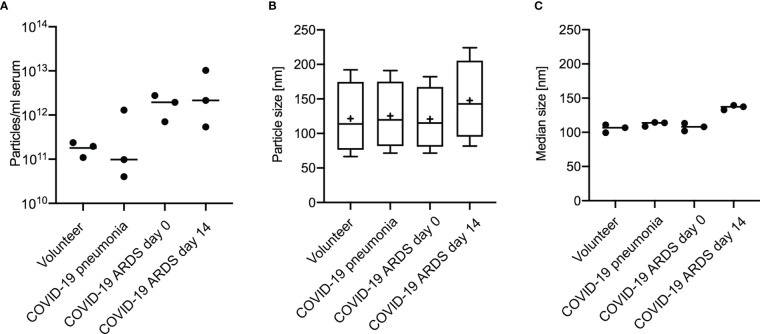
Analysis of extracellular vesicles by Nanoparticle Tracking Analysis shows the distribution of particle number **(A)**, particle size **(B)** and median particle size **(C)** according to subgroups in 3 individuals of each group. Plot A and B: Line = mean value, dot = modal value. Blot B: Boxplots, line=median, += modal value, Whiskers = quartiles.

For all groups spherical vesicle-like extracellular particles with a size of about 100 nm were detected by transmission electron microscopy (**Figure 2**). Particle concentrations for subgroups were 1.80E+11 ± 5.35E+10 particles/ml serum for volunteers, 4.77E+11 ± 5.77E+11 for patients with COVID-19 pneumonia, 1.80E+12 ± 8.43E+11 for COVID-19 ARDS patients at admission to the ICU, and 4.34E+12 ± 4.28E+12 in COVID-19 ARDS patients at day 14 of ICU treatment. The median particle diameter was 105.73 ± 4.74 nm for volunteers, 112.33 ± 2.58 nm for COVID-19 pneumonia patients, 107.80 ± 4.62 nm for patients with COVID-19 ARDS at admission, and 136.57 ± 2.72 nm for patients with COVID-19 ARDS at day 14 of ICU treatment (see **Figure 3** for a graphical representation).

### RNA Yield, Sequencing Quality and Mapping Distribution

There were large variations in total RNA yield between individual samples and different groups. As confirmed by Phred score levels, per base sequence quality of all libraries from all groups was high across all bases. Mean Phred scores were 38.42 ± 0.44 for volunteers, 38.33 ± 0.48 for COVID-19 pneumonia patients, 38.33 ± 0.44 for COVID-19 ARDS patients at ICU admission, and 38.46 ± 0.44 for COVID-19 ARDS patients at day 14 of ICU treatment. Total libraries added up in the average of 1.03E+E07 ± 5.71E+E06 reads for volunteers, 5.01E+E06 ± 3.61E+06 reads for patients with COVID-19 pneumonia, 5.34E+06 ± 3.55E+06 reads for those with COVID-19 ARDS at ICU admission, and 6.79E+06 ± 5.68E+06 reads for ARDS patients at day 14 of ICU treatment. When compared to COVID-19 pneumonia patients, library sizes were significantly higher for volunteers (p= 0.0163) and COVID-19 ARDS patients at admission to the ICU (p= 0.0274). miRNA precursors mapped to 24.99 ± 12.35% for volunteers, 9.89 ± 5.45 % for COVID-19 pneumonia patients, 16.99 ± 12.88% for patients with COVID-19 ARDS at d0, and 30.69 ± 14.79% for those with COVID-19 ARDS at day 14 of ICU treatment. miRNA mapping was significantly higher in volunteers (p=0.0027) as well as in COVID-19 ARDS patients at day 14 (p=0.0006), when compared to patients with COVID-19 pneumonia. For all other groups, no significant difference was detected for either total library size or miRNA mapping.

### Expression Levels of Significantly Regulated miRNAs According to Subgroups

**Figure 4:**
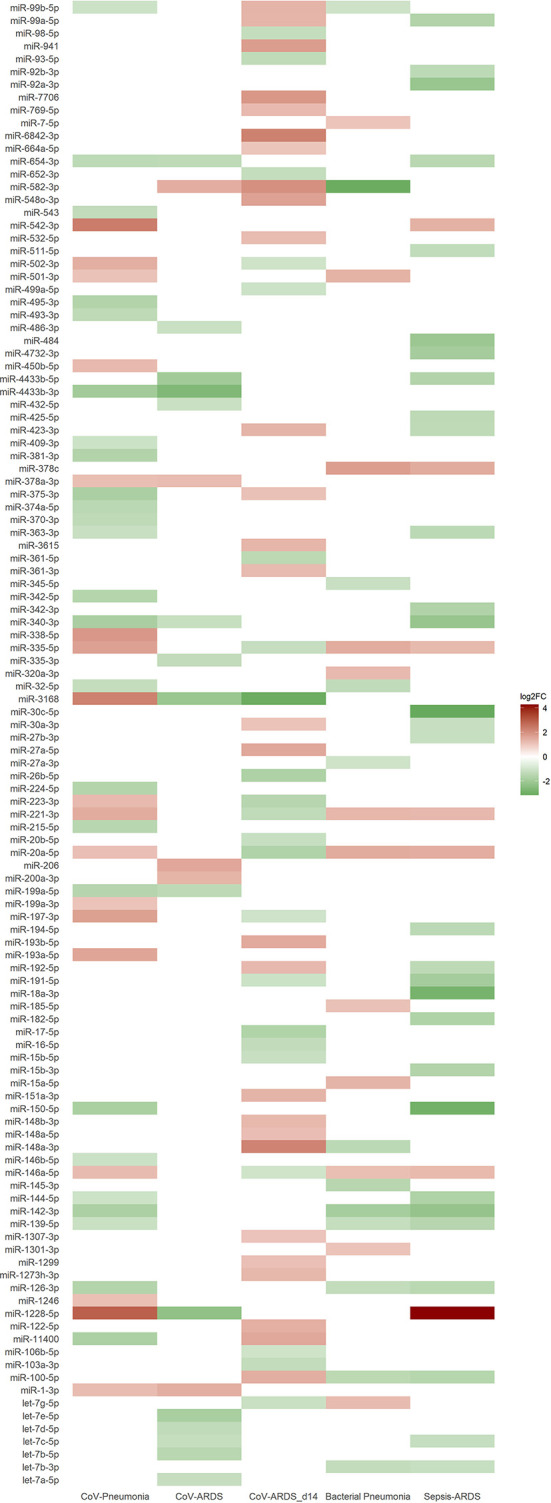
Heat map of differentially expressed miRNAs from small RNA sequencing based on the log2FC-value of the corresponding group comparison. Blank spaces indicate no significantly different expression between the groups of the respective comparison. Green values indicate a downregulation and red values an upregulation of miRNAs. Comparisons of the different patient groups: CoV-Pneumonia = healthy controls (baseline) compared to patients with COVID-19 pneumonia; CoV-ARDS = patients with COVID-19 pneumonia (baseline) compared to patients with COVID-19 ARDS; CoV-ARDS_d14 = patients with COVID-19 ARDS at admission to the ICU (baseline) compared to day 14 of ICU treatment; Bacterial Pneumonia = patients with bacterial community acquired pneumonia (baseline) compared to patients with COVID-19 pneumonia; Sepsis-ARDS = patients with sepsis on the day of ICU-admission (baseline) compared to patients with COVID-19 ARDS.

Small RNAseq revealed 43 significantly regulated EV-associated miRNAs (18 upregulated) between healthy controls and patients from the COVID-19 pneumonia group, and 20 (5 upregulated) between patients with COVID-19 pneumonia and patients with COVID-19 ARDS. When comparing patients with COVID-19 ARDS on day 0 and day 14 of their ICU stay, 50 miRNAs were differentially regulated (28 upregulated). For the comparison between patients with community acquired pneumonia and patients with COVID-19 pneumonia, 24 differentially regulated miRNAs were found (12 upregulated), and 37 miRNAs for the comparison between patients with sepsis of bacterial origin and patients with COVID-19 ARDS (7 upregulated). All significantly regulated miRNAs with base mean, log2FC, p and p-adjusted are listed in the **Supplementary e-Table 1**, differences in regulatory directions between groups are shown in **Figure 4**.

### Real Time Quantitative PCR Validation

Of 31 selected miRNAs from the RNAseq analysis, 17 showed the same regulatory direction and five met the cutoff of log2FC ≥ 1 or log2FC ≤ -1 and p ≤ 0.05 as applied for sequencing data. A possible explanation for miRNAs which failed in qPCR validation is that in contrast to small RNA sequencing starting with the cDNA fraction representing small RNA, only cDNA of total RNA can be used as input for qPCR. **Supplementary Tables e2, e3** show all results data of miRNA and mRNA qPCR validation.

### Signaling Networks in SARS-CoV-2 Infection and in Comparison to Community Acquired Pneumonia and Sepsis

**Figure 5:**
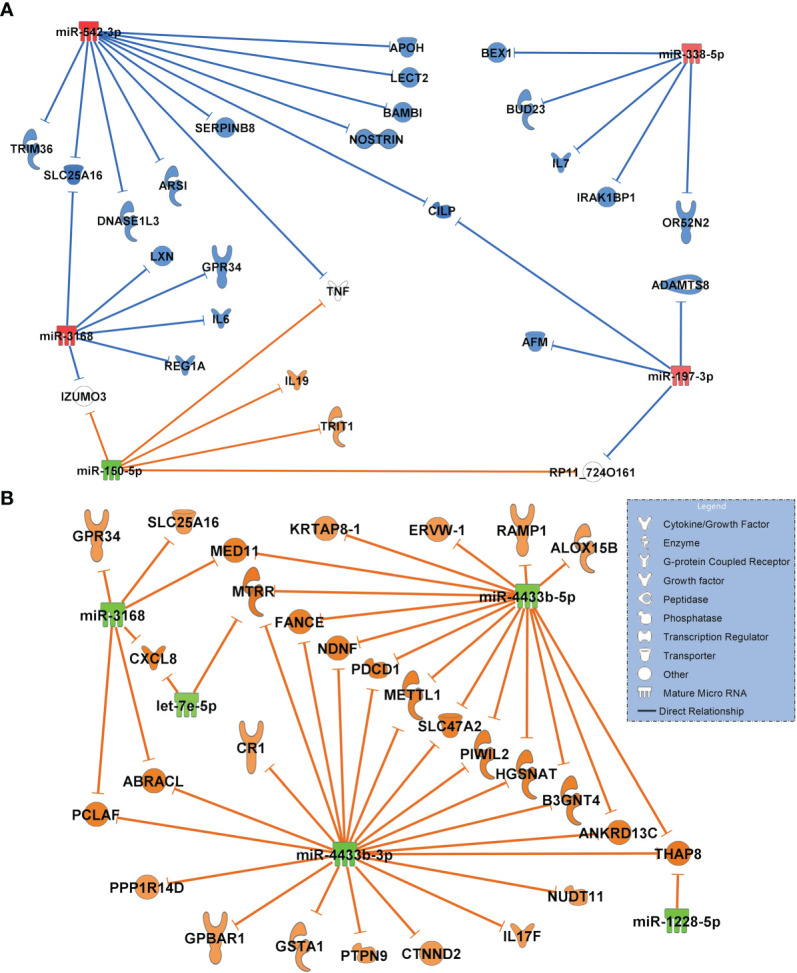
**(A)** Comparison of the highest differentially regulated miRNAs generated from small RNA sequencing data derived from circulating extracellular vesicles between healthy controls (n=18) and patients with COVID-19 pneumonia (n=15) and their predicted targets by *in-silico* analysis. Healthy controls were regarded as baseline for calculating log_2_FC and p-adjusted values. **(B)** Network of COVID-19 pneumonia patients (baseline) versus patients with COVID-19 ARDS (n=15). For identification of miRNAs, log2FC expression values were set to ≥ 1.8 or ≤-1.8 and only highly predicted or experimentally observed relationships are shown. Significantly upregulated miRNAs are presented in red and their predicted and presumably inhibited targets in blue. Downregulated miRNAs are shown in green, and their activated targets are depicted by orange colour. The legend inserted into the graph illustrates the type of target molecules according to the symbols used in the graph.

The network of healthy controls compared to COVID-19 pneumonia patients is shown in **Figure 5A**. This network includes only miRNAs with the greatest magnitude of change between groups (log2FC of ≥ ǀ1.8ǀ). miR-338-5p, miR-197-3p, miR-542-3p (all RT-qPCR-validated) were significantly upregulated targeting the majority of all transcripts in the network. miR-3168 was identified as a possible regulator of interleukin-6 (IL6) which was elevated in patients with COVID-19 pneumonia (25.1, IQR 15.2 - 88.2 pg/ml). miR-338-5p had inhibitory effects on OR52N2, an olfactory smell receptor in the nasal epithelium. Downregulated miR-150-5p was predicted to upregulate interleukin-19, an immunosuppressive cytokine. The network also showed an inhibition of targets by miR-542-3p with an anticoagulatory effect: SERPINB8, which promotes fibrinolysis and APOH, which inhibits coagulation factors.

The comparison of differentially regulated miRNAs and their predicted transcriptomic targets between patients with COVID-19 pneumonia and COVID-19 ARDS showed a network of five miRNAs (log2FC of ≥ ǀ1.8ǀ) which were all significantly downregulated (p_adjusted_<0.001) resulting in a fully activated network of all target molecules (**Figure 5B**). Upregulated CXCL8 (interleukin-8) was targeted by downregulated miR-3168 and let-7e-5p and also validated by RT-qPCR.

**Figure 6:**
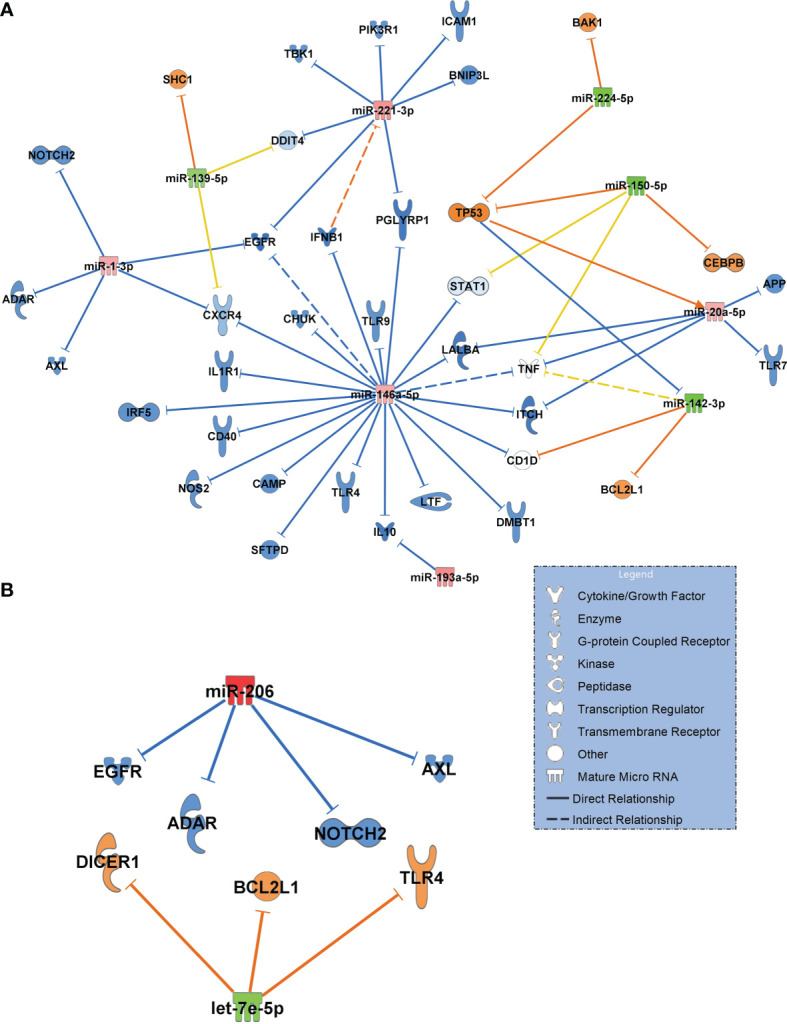
**(A)** Signalling network generated from small RNA sequencing data of healthy controls (n=18, baseline) in comparison to patients with COVID-19 pneumonia (n=15). **(B)** Network COVID-19 pneumonia (n=15, baseline) vs. COVID-19 ARDS (n=15). For generation of the networks, data were filtered for antimicrobial response and lung as an organ system. Only experimentally confirmed relationships between miRNAs and their targets were included. Significantly upregulated miRNAs are shown in red and their predicted and presumably inhibited targets in blue. Downregulated miRNAs are shown in green, and their activated targets are depicted by orange colour. Yellow lines and non–coloured molecules indicate undetermined regulatory effects because the transcripts were targeted by miRNAs with different directions of regulation. Solid lines indicate direct and dashed lines indirect relationships. The legend inserted into the graph illustrates the type of target molecules according to the symbols used in the graph.

Next, we constructed networks of miRNAs focusing on antimicrobial response and the lung as an organ target for the healthy controls and COVID-19 pneumonia patients. **Figure 6A** shows the resulting network. Important findings included miR-1-3p targeting the alpha chemokine receptor CXCR4 and miR-193a-5p inhibiting interleukin-10 (IL10), which were validated in this network. Upregulated miR-146a-5p was identified to target and to downregulate toll-like receptor 4 (TLR4).

The comparison between COVID-19 pneumonia and COVID-19 ARDS (also filtered for antimicrobial response and lung as a target organ) identified upregulated miR-206 (validated) and the downregulated let-7e-5p (**Figure 6B**). In contrast to the healthy control – COVID-19 pneumonia network (**Figure 6A**), TLR4 was activated by downregulated let-7e-5p in the pneumonia–ARDS network.

**Figure 7:**
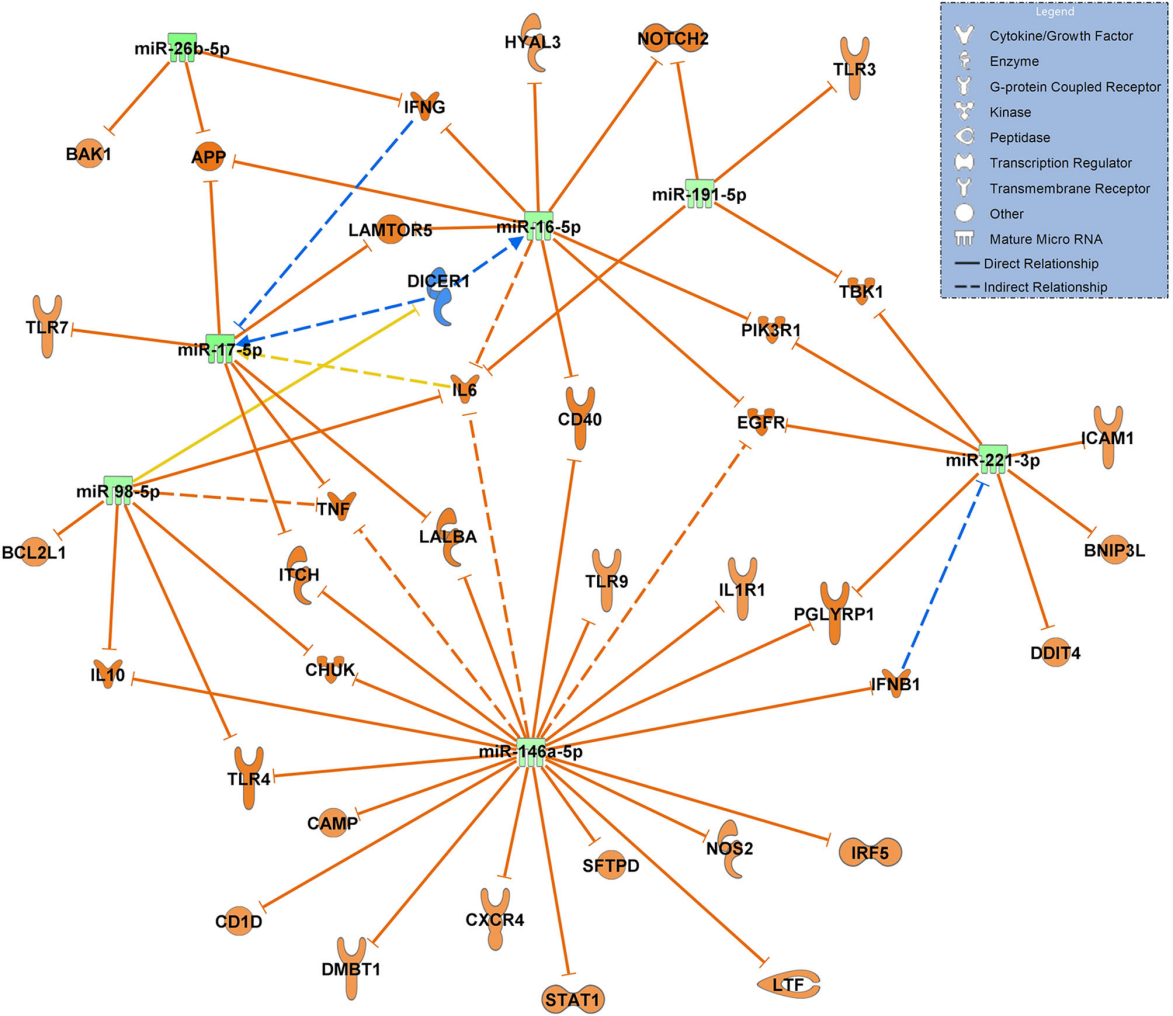
Changes in miRNA signalling networks during ICU treatment of patients with COVID-19 ARDS based on small RNA sequencing data. miRNA expression values between day 0 (admittance to the ICU, baseline, n=15) and day 14 of ICU treatment (n=13) were compared. All miRNAs in the network were downregulated represented by green colouring and their presumed targets by orange colour. Yellow lines indicate undetermined regulatory effects because the transcripts were targeted by miRNAs with different directions of regulation. Solid lines indicate direct and dashed lines indirect relationships. Data from small RNA sequencing were filtered for antimicrobial response and lung as an organ system and confidence levels were set to experimentally observed. The legend inserted into the graph illustrates the type of target molecules according to the symbols used in the graph.

When constructing a network filtered for antimicrobial response and lung in COVID-19 ARDS on admission to the ICU vs. day 14, all 7 miRNAs in this network were significantly downregulated suggesting a ubiquitous activation of the network. TLR4 remained activated at day 14 of ICU therapy (targeted by miR-98-5p and miR-146a-5p). miR-17-5p, miR-191-5p (both targeting IL6) and miR-221-3p (targeting ICAM1 among others) were validated in the network (**Figure 7**).

**Figure 8:**
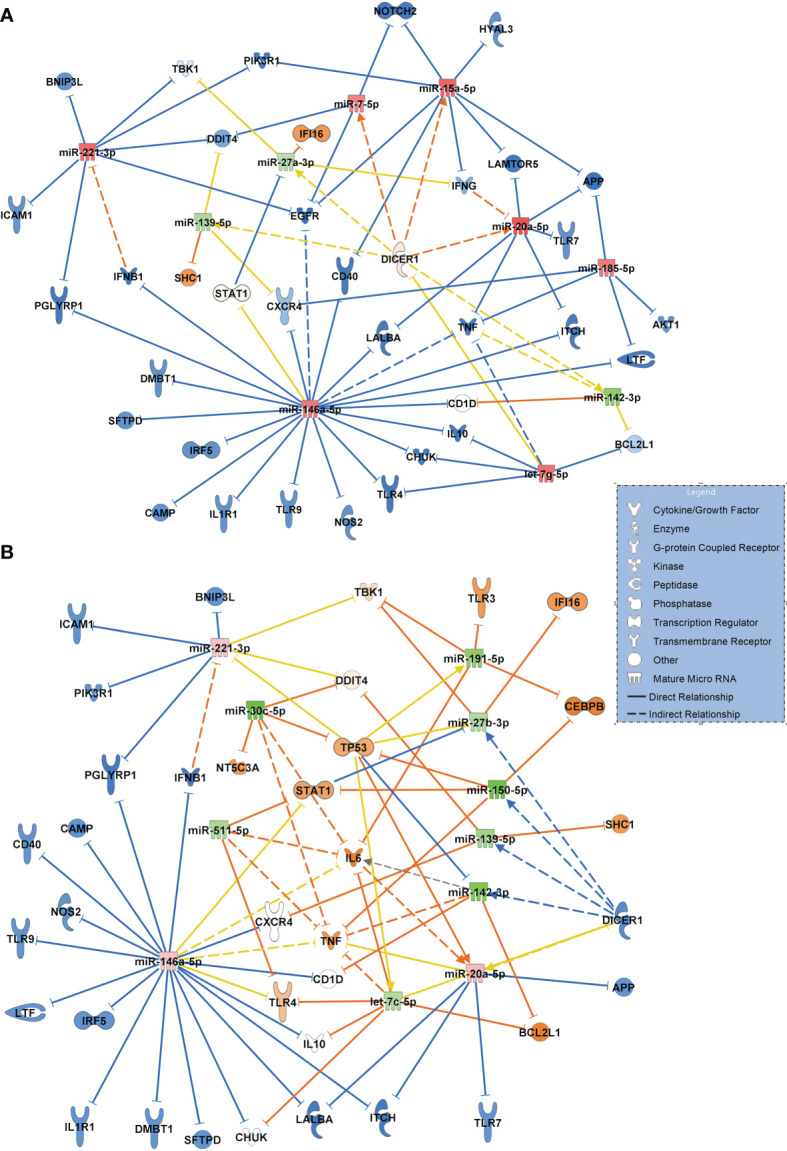
**(A)** Signaling network comparing miRNA expression values and their target regulation between patients with community acquired pneumonia of bacterial origin (n=12, baseline) and patients with COVID-19 pneumonia (n=15). **(B)** Network resulting from a comparison between patients with sepsis induced lung injury of bacterial origin (n=28, baseline) and patients with COVID-19 ARDS (n=15) at admission to the ICU (day 0 of treatment). Downregulated miRNAs are shown in green and the corresponding mRNA targets in orange colour. Upregulated miRNAs are marked in red and their inhibited targets in blue. For miRNA target scanning, data were filtered for antimicrobial response and lung as an organ system and confidence levels were set to experimentally observed. Yellow lines and non-coloured molecules indicate undetermined regulatory effects because the transcripts were targeted by miRNAs with different directions of regulation. Solid lines indicate direct and dashed lines indirect relationships. The legend inserted into the graph illustrates the type of target molecules according to the symbols used in the graph.

**Figure 8A** visualizes the comparison between patients with community acquired pneumonia and patients with COVID-19 pneumonia, using the same filter criteria *antimicrobial response* and *lung* as a target organ. Seven upregulated miRNAs (miR-185-5p and let-7g-5p were validated) and 3 downregulated miRNAs (miR-27a-3p validated) resulted in a mostly inactivated network with a predicted downregulation of several immunoreactive molecules including TLR4, TLR9 and TLR7, ICAM1, TNF and the inflammatory molecule PIK3R1 (validated and targeted by miR-221-3p and miR-15a-5p) suggesting a general immunosuppressive effect of SARS-CoV-2.

The network for patients with sepsis induced ARDS compared to patients with COVID-19 ARDS resulted in a more complex interaction shown in **Figure 8B**. Eight downregulated miRNAs (of which miR-511-5p, miR-30c-5p, miR-27b-3p, miR-150-5p and miR-139-5p were validated) and 3 upregulated miRNAs resulted in a partially activated network which included TLR4, IL6, TP53 and STAT1 suggesting a central role for these transcripts in COVID-19 induced lung injury.

All sequencing data for the networks including log2FC, p and p-adjusted values are presented in e-table 1 of the **Supplementary e-Table 1**. RT-qPCR data are shown in the **Supplementary e-Tables 2, 3**.

## Discussion

In our study, we analyzed miRNAs derived from extracellular vesicles in patients with SARS-CoV-2 infection and compared the expression patterns of significantly regulated miRNAs between healthy individuals, patients with COVID-19 pneumonia and community acquired pneumonia of bacterial origin. COVID-19 ARDS as a severe manifestation of SARS-CoV-2 infection was then compared to ARDS as a complication of bacterial sepsis. These analyses were performed to delineate some of the molecular mechanisms underlying the remarkable clinical differences in disease severity and outcome between these disorders and to give early suggestions for future biomarkers.

The first group we analyzed were healthy controls in comparison to patients with COVID-19 pneumonia. When focusing on maximally regulated miRNAs, miR-338-5p, miR-197-3p, miR-542-3p were identified as important regulatory molecules. Of particular interest in this network appeared miR-338-5p (RT-pPCR validated) targeting IL6, a key mediator of the cytokine storm seen in severe cases of COVID-19 (25) and a pharmacologic target of tocilizumab, which may provide a survival benefit in COVID-19 patients (26). miR-338-5p also inhibited the expression of the smell receptor OR52N2 located in the nasal mucosa. This finding could help to explain the hyp- or anosmia often seen in the early phase of SARS-CoV-2 infection (27). More importantly, olfactory cells are regarded as an entry gate of the virus for neuroinvasiveness, although this effect has previously mainly been ascribed to the ACE2 receptor (28). Another interesting finding from this network is the inhibition of two transcripts with coagulatory effects (exosome-related APOH (29) and SERPINB8 (30) by strongly upregulated miR-542-3p. This could be a possible additional risk factor for thromboembolic complications seen in nearly 30% of COVID-19 patients (31). SERPINB8 (serpine petidase inihibitor) is also a strong inhibitor of furin (30), a cleaving protein, and SERPINB8 inhibition by miR-542-3p is predicted to result in increased furin activity. Binding of the SARS-CoV-2 on its cellular ACE2 receptor is a prerequisite for cellular entry requires cleavage of the viral spike protein by furin. It has been suggested that the insertion of a unique cleaving site for furin into the spike protein of SARS-CoV-2 which is absent in earlier appearing lineages of CoVs is responsible for its pronounced infectivity and transmissibility (32). This suggests an important role for the miR-542-3p - SERPINB8 pathway for the pathogenesis of COVID-19 seen in the network.

In contrast to the network generated for the healthy state versus COVID-19 pneumonia, all significantly regulated miRNAs from the corresponding analysis comparing patients with COVID-19 pneumonia to those who had progressed to the severe acute respiratory corona virus-2 syndrome (ARDS) were downregulated suggesting a fully activated network of all transcripts. The proinflammatory cytokine CXCL8 (Interleukin-8, RT-qPCR validated), a regulator of neutrophil recruitment into the lung causing epithelial damage in COVID-19 (33), was targeted by miR-3168 and let-7e-5p (both downregulated) in this network. CXCL8 is known to be highly expressed in patients with severe COVID-19 disease (ARDS) but not in individuals with a mild disorder (34).

When filtering for *antimicrobial response* and *lung* as a target organ to get a more complete and specific comparison between the healthy state and COVID-19 pneumonia, many of the identified transcripts were downregulated in the network and are important for the defense against viral infections and antibody production (such as ADAR, CAMP, IFNB1, IRF5, LTF, SFTPD, TLR4, TLR7, TLR9, IL10 and CD40) which indicates an inhibition of the innate immune system by the virus. This could also increase the risk for bacterial or fungal superinfection in COVID-19. An immunoparalysis despite high levels of proinflammatory cytokines has also been described earlier (35) and is in accordance with our findings.

It is also of interest that the network comparing COVID-19 pneumonia to COVID-19 ARDS appears to be far less complex than the corresponding comparison between the healthy state and COVID-19 pneumonia with only two miRNAs differentially regulated between both groups. In contrast, when patients with community acquired pneumonia were compared to individuals with ARDS as a complication of bacterial pneumonia in a recent study, 25 cell free miRNAs were differentially regulated between the groups (9). This suggests that the difference in pathology between early pneumonia and the later development of severe pulmonary failure (ARDS) in COVID-19 is more quantitative than qualitative and that the virus causes diffuse and uncontrolled lung injury from the beginning of the infection. This is also corroborated by the observation that no pathogen was detected in most samples derived from patients with community acquired pneumonia and progression to ARDS was comparatively rare whereas ARDS was seen in 47% of patients presenting with COVID-19 pneumonia and a presumable persisting SARS-CoV-2 infection. This may also reflect the fact that for bacterial community acquired pneumonia effective antibiotics exist whereas no specific antiviral therapy was available for COVID-19 patients.

An interesting role in both networks is played by TLR4. The comparison of the regulatory direction of TLR4 in COVID-19 pneumonia to COVID-19 ARDS showed inhibition of TLR4 by miR-146a-5p in pneumonia and upregulation in ARDS by downregulated let-7e-5p. The let-7e cluster was shown to be an effective negative regulator of TLR4 signaling in human monocytes during an inflammatory stimulus (LPS) (36). Activated TLR4 is also a strong binding partner of the SARS-CoV-2 spike protein that increases cell surface ACE2 expression to facilitate viral entry. This results in the destruction of type-II alveolar cells that secrete pulmonary surfactants, pulmonary-capillary leakage and ARDS (37, 38). This finding could help to explain differences in the clinical course of patients presenting with pneumonia who recover without the progression to pulmonary failure and ARDS.

The network illustrating the response to bacterial community acquired pneumonia in comparison to SARS-CoV-2 infection suggested a strong immunosuppressive effect of the virus with a presumed downregulation of several immunoreactive molecules including TLR9 and TLR7, ICAM1, TNF and the inflammatory molecule PIK3R1 (validated and targeted by miR-221-3p and miR-15a-5p) pointing to a general immunosuppressive effect of SARS-CoV-2.

The network constructed for comparing patients with sepsis to patients with COVID-19 ARDS also suggested an upregulation of several key molecules relevant for the pathogenesis of COVID-19 ARDS which included TLR4, IL6, and - in particular - STAT1 (signal transducer and activator of transcription 1). It has been suggested that viral components of SARS-CoV-2 induce an activation of STAT1 and plasminogen activator inhibitor-1 leading to coagulopathy, intravascular thrombosis and a further activation of TLR4 (39). TLR4 was activated in the network by downregulated let-7c-5p. The comparison of molecular signaling in the network to that of sepsis-associated ARDS also suggests that these signaling cascades are specific to COVID-19 ARDS and probably less common in bacterial sepsis. TLR4 was also identified as a potential target of miR-146a-5p. This EV associated RNA was significantly upregulated in COVID-19 pneumonia and COVID -19 ARDS in comparison to healthy controls, non-COVID bacterial pneumonia and to non-COVID sepsis induced ARDS and downregulated when day 14 of COVID-19 ARDS treatment was compared to ICU admittance (day 0). This suggests a specific change in expression values of this miRNA in the presence of a virulent virus. In fact, miR-146a was identified in circulating EVs (exosomes) (40), its upregulation after HCV infection was shown to promote the assembly of virus particles and their release from infected cells (41) and that the SARS-CoV-2 genome is able to hijack miRNAs from the miR-146 group to modulate the immune response by downstream effects on their target mRNAs (42).

One has to keep in mind, however, that the signaling networks comparing community acquired pneumonia to COVID-19 pneumonia and sepsis induced ARDS to COVID-19 ARDS were based on a previously acquired small RNA sequencing data set (9). This could lead to differences in expression values of miRNAs and unquantified matrix effects, which are difficult to control when the signaling networks were constructed and limit the comparability between groups. Furthermore, as the miRNAs analyzed in our study were derived from circulating serum extracellular vesicle precipitates, one should be aware of the fact that these precipitates contain a significant amount of cell-free proteins, which might include well-known carriers of circulating miRNAs such as argonaute 2 or lipoprotein particles (43, 44). Several miRNAs characterized in our study may therefore be co-precipitated from serum and not be exclusively derived from extracellular vesicles. Further studies in this field will require additional validation using more vesicle-specific isolation methods such as density gradient centrifugation (45).

Despite these limitations, EV-associated miRNAs and their targeted cellular transcripts were shown to have an important regulatory function in the immune response to SARS-CoV-2 and the progression to severe disease and may serve as biomarker candidates or targets for pharmacologic interventions.

## Data Availability Statement

The data presented in the study are deposited in the European Nucleotide Archive (ENA) repository, accession number PRJEB49135.

## Ethics Statement

Ethics approval for the study was obtained from the Ethics Committee of the Medical Faculty of the Ludwig-Maximilians-University of Munich under protocols #551-14 and 20-299. The patients/participants provided their written informed consent to participate in this study.

## Author Contributions

All authors contributed to the study conception and design. Material preparation and data collection was performed by FB, AM, MK, and EA. Molecular analysis was executed by AL, MR, and SH. Data analysis was done by DB, FB, BK, and J-NB. AM, GS, and MP worked on the manuscript. All authors commented on previous versions of the manuscript. All authors contributed to the article and approved the submitted version.

## Funding

The study was supported by the Bavarian Research Foundation under protocol #AZ-1439-20C and by the TUM Universitätsstiftung.

## Conflict of Interest

JNB is employed by QIAGEN, Redwood, CA, USA and QIAGEN products were used in the study.

The remaining authors declare that the research was conducted in the absence of any commercial or financial relationships that could be construed as a potential conflict of interest.

## Publisher’s Note

All claims expressed in this article are solely those of the authors and do not necessarily represent those of their affiliated organizations, or those of the publisher, the editors and the reviewers. Any product that may be evaluated in this article, or claim that may be made by its manufacturer, is not guaranteed or endorsed by the publisher.
