# Supplementary material for: Extracellular Vesicle Associated miRNAs Regulate Signaling Pathways Involved in COVID-19 Pneumonia and the Progression to Severe Acute Respiratory Corona Virus-2 Syndrome
Source: Front Immunol. 2021 Dec 9;12:784028. doi: 10.3389/fimmu.2021.784028 (PMC8696174; doi:10.3389/fimmu.2021.784028)
Supplement: Supplementary file 1 [file DataSheet_1.zip › Supplement Meidert et al/e-Table 1 NGS details.docx]

e-Table 1: Differentially regulated miRNAs between groups.

| **Controls vs. COVID-19 pneumonia** | **miRNA** | **baseMean** | **log2FC** | **P** | **p_adjusted_** | **Mean controls** | **Mean COVID-19 pneumonia** |
| --- | --- | --- | --- | --- | --- | --- | --- |
|  | *miR-1-3p* | *1721.36* | *1.204* | *0.004* | *0.021* | *471.71* | *1221.91* |
|  | miR-11400 | 127.63 | -1.744 | <0.001 | 0.001 | 247.02 | 75.22 |
|  | miR-1228-5p | 290.03 | 2.916 | <0.001 | <0.001 | 99.39 | 673.63 |
|  | miR-1246 | 88.54 | 1.136 | <0.001 | 0.002 | 47.79 | 91.48 |
|  | miR-126-3p | 12751.85 | -1.580 | <0.001 | <0.001 | 23241.22 | 7441.86 |
|  | *miR-139-5p* | *325.79* | *-1.136* | *<0.001* | *<0.001* | *522.15* | *218.84* |
|  | *miR-142-3p* | *347.50* | *-1.743* | *<0.001* | *<0.001* | *660.17* | *195.81* |
|  | miR-144-5p | 77.11 | -1.041 | 0.001 | 0.007 | 122.68 | 53.53 |
|  | *miR-146a-5p* | *5600.33* | *1.226* | *<0.001* | *<0.001* | *3207.44* | *6995.46* |
|  | miR-146b-5p | 996.32 | -1.093 | <0.001 | <0.001 | 1479.96 | 665.85 |
|  | *miR-150-5p* | *89.57* | *-1.783* | *<0.001* | *<0.001* | *165.25* | *40.56* |
|  | *miR-193a-5p* | *605.71* | *1.624* | *<0.001* | *<0.001* | *200.11* | *648.24* |
|  | *miR-197-3p* | *167.95* | *1.707* | *<0.001* | *<0.001* | *70.98* | *254.68* |
|  | miR-199a-3p | 1227.23 | 1.068 | <0.001 | <0.001 | 711.45 | 1420.90 |
|  | miR-199a-5p | 247.07 | -1.543 | <0.001 | 0.001 | 474.85 | 155.30 |
|  | *miR-20a-5p* | *1239.26* | *1.161* | *<0.001* | *<0.001* | *575.62* | *1306.31* |
|  | miR-215-5p | 164.33 | -1.495 | <0.001 | <0.001 | 295.20 | 105.45 |
|  | *miR-221-3p* | *1288.16* | *1.521* | *<0.001* | *<0.001* | *529.36* | *1478.28* |
|  | miR-223-3p | 360.82 | 1.237 | <0.001 | 0.002 | 207.58 | 471.03 |
|  | *miR-224-5p* | *108.49* | *-1.572* | *<0.001* | *0.001* | *187.92* | *74.14* |
|  | *miR-3168* | *2044.41* | *2.278* | *<0.001* | *<0.001* | *919.18* | *4484.82* |
|  | miR-32-5p | 121.91 | -1.252 | 0.001 | 0.007 | 186.28 | 84.70 |
|  | miR-335-5p | 168.06 | 1.695 | <0.001 | <0.001 | 73.13 | 207.41 |
|  | *miR-338-5p* | *153.15* | *1.877* | *<0.001* | *<0.001* | *49.51* | *183.92* |
|  | miR-340-3p | 133.44 | -1.798 | <0.001 | <0.001 | 260.46 | 75.06 |
|  | miR-342-5p | 153.37 | -1.561 | <0.001 | <0.001 | 292.53 | 86.89 |
|  | miR-363-3p | 965.26 | -1.162 | 0.001 | 0.005 | 1610.42 | 654.94 |
|  | miR-370-3p | 305.35 | -1.364 | 0.001 | 0.007 | 532.46 | 216.04 |
|  | miR-374a-5p | 178.68 | -1.425 | <0.001 | <0.001 | 323.27 | 110.43 |
|  | miR-375-3p | 449.03 | -1.777 | <0.001 | <0.001 | 761.66 | 263.39 |
|  | miR-378a-3p | 1298.11 | 1.167 | <0.001 | <0.001 | 488.33 | 1069.26 |
|  | miR-381-3p | 254.22 | -1.592 | <0.001 | 0.002 | 459.25 | 152.06 |
|  | miR-409-3p | 301.92 | -1.071 | 0.009 | 0.040 | 513.54 | 232.89 |
|  | miR-4433b-3p | 336.68 | -1.932 | 0.001 | 0.005 | 692.54 | 207.38 |
|  | miR-450b-5p | 95.00 | 1.240 | <0.001 | 0.001 | 36.63 | 94.91 |
|  | miR-493-3p | 81.75 | -1.373 | 0.004 | 0.021 | 135.25 | 54.28 |
|  | miR-495-3p | 51.45 | -1.598 | <0.001 | 0.002 | 95.54 | 28.68 |
|  | miR-501-3p | 168.28 | 1.095 | <0.001 | <0.001 | 91.66 | 184.04 |
|  | miR-502-3p | 84.99 | 1.437 | <0.001 | <0.001 | 32.15 | 89.28 |
|  | *miR-542-3p* | *83.16* | *2.378* | *<0.001* | *<0.001* | *16.95* | *96.43* |
|  | miR-543 | 256.91 | -1.281 | 0.002 | 0.011 | 440.29 | 190.59 |
|  | miR-654-3p | 98.14 | -1.384 | <0.001 | 0.002 | 179.21 | 71.72 |
|  | miR-99b-5p | 430.31 | -1.068 | <0.001 | <0.001 | 643.98 | 283.27 |
| **Covid-19 pneumonia vs. COVID-19 ARDS** | **miRNA** | **baseMean** | **log2FC** | **P** | **p_adjusted_** | **Mean COVID-19 pneumonia** | **Mean COVID-19 ARDS** |
|  | let-7a-5p | 18960.33 | -1.203 | <0.001 | <0.001 | 23301.52 | 10803.74 |
|  | let-7b-5p | 15533.13 | -1.480 | <0.001 | <0.001 | 17264.99 | 6211.69 |
|  | let-7c-5p | 232.88 | -1.224 | <0.001 | 0.001 | 269.44 | 119.08 |
|  | let-7d-5p | 1456.89 | -1.321 | <0.001 | <0.001 | 1723.17 | 670.61 |
|  | *let-7e-5p* | *199.74* | *-1.808* | *<0.001* | *<0.001* | *184.38* | *54.65* |
|  | miR-1-3p | 1721.36 | 1.437 | 0.001 | 0.009 | 1221.91 | 3720.39 |
|  | *miR-1228-5p* | *290.03* | *-2.286* | *<0.001* | *0.006* | *673.63* | *135.21* |
|  | miR-199a-5p | 247.07 | -1.393 | <0.001 | 0.006 | 155.30 | 65.51 |
|  | miR-200a-3p | 95.41 | 1.338 | 0.006 | 0.042 | 69.12 | 130.11 |
|  | *miR-206* | *150.84* | *1.578* | *0.005* | *0.036* | *101.32* | *277.46* |
|  | *miR-3168* | *2044.41* | *-2.126* | *<0.001* | *0.003* | *4484.82* | *954.26* |
|  | miR-335-3p | 158.81 | -1.284 | 0.001 | 0.012 | 151.24 | 65.01 |
|  | miR-340-3p | 133.44 | -1.196 | 0.001 | 0.012 | 75.06 | 39.40 |
|  | miR-378a-3p | 1298.11 | 1.204 | <0.001 | 0.001 | 1069.26 | 2498.68 |
|  | miR-432-5p | 165.08 | -1.134 | 0.006 | 0.041 | 143.90 | 66.14 |
|  | *miR-4433b-3p* | *336.68* | *-2.630* | *<0.001* | *<0.001* | *207.38* | *38.95* |
|  | *miR-4433b-5p* | *78.51* | *-1.971* | *<0.001* | *<0.001* | *85.52* | *21.72* |
|  | miR-486-3p | 694.93 | -1.138 | <0.001 | 0.003 | 1054.39 | 506.50 |
|  | miR-582-3p | 207.03 | 1.460 | <0.001 | 0.003 | 172.33 | 312.63 |
|  | miR-654-3p | 98.14 | -1.367 | <0.001 | 0.006 | 71.72 | 27.29 |
| **COVID-19 ARDS admission to ICU vs. day 14** | **miRNA** | **baseMean** | **log2FC** | **P** | **p_adjusted_** | **Mean ICU admission** | **Mean day 14** |
|  | let-7g-5p | 8394.01 | -1.139 | <0.001 | <0.001 | 12390.25 | 6200.67 |
|  | miR-100-5p | 455.64 | 1.461 | <0.001 | <0.001 | 276.40 | 620.83 |
|  | miR-103a-3p | 913.85 | -1.257 | <0.001 | <0.001 | 1425.42 | 628.04 |
|  | miR-106b-5p | 137.15 | -1.038 | <0.001 | <0.001 | 190.08 | 89.56 |
|  | miR-11400 | 67.29 | 1.568 | <0.001 | 0.002 | 35.38 | 107.56 |
|  | miR-122-5p | 54580.68 | 1.423 | <0.001 | 0.001 | 25313.74 | 88608.89 |
|  | miR-1273h-3p | 122.87 | 1.298 | <0.001 | <0.001 | 72.70 | 154.14 |
|  | miR-1299 | 72.75 | 1.127 | 0.001 | 0.006 | 45.17 | 83.02 |
|  | miR-1307-3p | 448.78 | 1.066 | <0.001 | 0.001 | 278.63 | 599.63 |
|  | *miR-146a-5p* | *4885.51* | *-1.011* | *<0.001* | *0.001* | *6694.42* | *3623.28* |
|  | *miR-148a-3p* | *367089.70* | *2.229* | *0.007* | *0.035* | *137152.64* | *771999.02* |
|  | miR-148a-5p | 92.65 | 1.175 | <0.001 | <0.001 | 54.51 | 113.44 |
|  | miR-148b-3p | 1233.68 | 1.280 | <0.001 | <0.001 | 736.00 | 2062.06 |
|  | miR-151a-3p | 17259.05 | 1.353 | <0.001 | <0.001 | 10854.18 | 30963.74 |
|  | miR-15b-5p | 110.15 | -1.167 | <0.001 | 0.001 | 152.86 | 68.93 |
|  | *miR-16-5p* | *1000.69* | *-1.279* | *<0.001* | *<0.001* | *1506.85* | *688.16* |
|  | *miR-17-5p* | *342.95* | *-1.642* | *<0.001* | *<0.001* | *558.84* | *188.60* |
|  | *miR-191-5p* | *2158.43* | *-1.078* | *<0.001* | *<0.001* | *3185.62* | *1567.46* |
|  | miR-192-5p | 2810.77 | 1.248 | <0.001 | <0.001 | 1710.64 | 3951.04 |
|  | miR-193b-5p | 72.35 | 1.538 | <0.001 | 0.001 | 25.95 | 158.20 |
|  | miR-197-3p | 167.27 | -1.021 | 0.002 | 0.013 | 191.53 | 104.80 |
|  | miR-20a-5p | 1206.46 | -1.638 | <0.001 | <0.001 | 1876.85 | 680.94 |
|  | miR-20b-5p | 66.01 | -1.194 | 0.001 | 0.006 | 111.28 | 50.98 |
|  | *miR-221-3p* | *1322.09* | *-1.325* | *<0.001* | *<0.001* | *1895.45* | *872.51* |
|  | miR-223-3p | 276.93 | -1.517 | <0.001 | <0.001 | 421.47 | 164.95 |
|  | *miR-26b-5p* | *1502.37* | *-1.690* | *<0.001* | *<0.001* | *2618.08* | *878.35* |
|  | miR-27a-5p | 211.15 | 1.571 | <0.001 | 0.002 | 118.69 | 345.31 |
|  | miR-30a-3p | 92.88 | 1.072 | 0.001 | 0.006 | 65.48 | 124.91 |
|  | miR-3168 | 466.71 | -3.042 | <0.001 | <0.001 | 882.06 | 263.36 |
|  | miR-335-5p | 145.98 | -1.208 | <0.001 | 0.002 | 223.57 | 99.87 |
|  | miR-361-3p | 318.73 | 1.221 | <0.001 | <0.001 | 206.00 | 460.25 |
|  | miR-361-5p | 166.55 | -1.416 | <0.001 | <0.001 | 208.25 | 84.59 |
|  | miR-3615 | 3945.01 | 1.304 | <0.001 | <0.001 | 2165.30 | 6708.56 |
|  | miR-375-3p | 325.29 | 1.114 | 0.002 | 0.015 | 247.75 | 416.05 |
|  | miR-423-3p | 2893.57 | 1.339 | <0.001 | <0.001 | 1853.62 | 3868.68 |
|  | miR-499a-5p | 371.31 | -1.076 | 0.003 | 0.016 | 388.05 | 197.19 |
|  | miR-502-3p | 96.48 | -1.040 | <0.001 | <0.001 | 136.62 | 68.72 |
|  | miR-532-5p | 503.75 | 1.205 | <0.001 | <0.001 | 326.93 | 686.15 |
|  | miR-548o-3p | 139.63 | 1.676 | <0.001 | <0.001 | 68.14 | 257.02 |
|  | miR-582-3p | 826.10 | 2.009 | <0.001 | <0.001 | 301.69 | 1909.46 |
|  | miR-652-3p | 53.94 | -1.243 | <0.001 | <0.001 | 82.30 | 33.61 |
|  | miR-664a-5p | 62.91 | 1.065 | <0.001 | <0.001 | 45.01 | 104.27 |
|  | miR-6842-3p | 93.11 | 2.276 | <0.001 | <0.001 | 35.18 | 195.43 |
|  | miR-769-5p | 82.22 | 1.233 | <0.001 | 0.001 | 49.29 | 119.40 |
|  | miR-7706 | 122.43 | 1.879 | <0.001 | <0.001 | 60.47 | 247.19 |
|  | miR-93-5p | 667.71 | -1.342 | <0.001 | <0.001 | 933.13 | 382.11 |
|  | miR-941 | 1025.17 | 1.768 | <0.001 | <0.001 | 532.59 | 1882.93 |
|  | *miR-98-5p* | *225.61* | *-1.252* | *<0.001* | *<0.001* | *370.60* | *144.99* |
|  | miR-99a-5p | 14102.34 | 1.321 | <0.001 | <0.001 | 7945.07 | 20630.07 |
|  | miR-99b-5p | 479.90 | 1.353 | <0.001 | <0.001 | 306.01 | 762.60 |
| **Community acquired pneumonia vs. COVID-19 pneumonia** | **miRNA** | **baseMean** | **log2FC** | **P** | **p_adjusted_** | **Mean community acquired pneumonia** | **Mean COVID-19 pneumonia** |
|  | hsa-let-7b-3p | 103.09 | -1.257 | 0.001 | 0.019 | 164.51 | 60.66 |
|  | *hsa-let-7g-5p* | *11462.74* | *1.214* | *<0.001* | *0.002* | *6574.09* | *18511.18* |
|  | hsa-miR-100-5p | 965.76 | -1.409 | 0.002 | 0.042 | 1166.50 | 244.72 |
|  | hsa-miR-126-3p | 18273.75 | -1.272 | <0.001 | 0.009 | 18722.98 | 10202.70 |
|  | hsa-miR-1301-3p | 98.96 | 1.077 | 0.002 | 0.032 | 80.74 | 157.93 |
|  | *hsa-miR-139-5p* | *555.23* | *-1.221* | *<0.001* | *0.013* | *626.13* | *304.54* |
|  | *hsa-miR-142-3p* | *302.92* | *-1.866* | *<0.001* | *0.002* | *216.06* | *267.58* |
|  | hsa-miR-145-3p | 308.10 | -1.500 | <0.001 | 0.002 | 435.64 | 223.38 |
|  | *hsa-miR-146a-5p* | *5987.70* | *1.168* | *0.002* | *0.032* | *4814.30* | *9752.22* |
|  | hsa-miR-148a-3p | 199496.63 | -1.405 | 0.002 | 0.032 | 256768.34 | 112932.62 |
|  | *hsa-miR-15a-5p* | *64.15* | *1.343* | *0.001* | *0.030* | *30.57* | *118.45* |
|  | *hsa-miR-185-5p* | *10151.92* | *1.116* | *0.002* | *0.035* | *9264.08* | *12500.94* |
|  | *hsa-miR-20a-5p* | *1027.46* | *1.512* | *<0.001* | *0.003* | *387.32* | *1821.20* |
|  | *hsa-miR-221-3p* | *1458.85* | *1.288* | *0.002* | *0.032* | *1084.82* | *2071.73* |
|  | *hsa-miR-27a-3p* | *15168.51* | *-1.037* | *0.001* | *0.030* | *15930.56* | *19576.10* |
|  | hsa-miR-32-5p | 125.89 | -1.316 | 0.001 | 0.026 | 110.82 | 115.96 |
|  | hsa-miR-320a-3p | 15070.38 | 1.238 | <0.001 | 0.009 | 18197.45 | 18534.94 |
|  | hsa-miR-335-5p | 152.03 | 1.498 | 0.001 | 0.030 | 82.32 | 288.97 |
|  | hsa-miR-345-5p | 100.74 | -1.158 | 0.001 | 0.019 | 104.42 | 67.46 |
|  | hsa-miR-378c | 54.09 | 1.757 | <0.001 | 0.009 | 39.81 | 58.99 |
|  | hsa-miR-501-3p | 303.96 | 1.372 | <0.001 | 0.005 | 217.90 | 256.95 |
|  | hsa-miR-582-3p | 393.39 | -3.064 | <0.001 | <0.001 | 913.62 | 240.39 |
|  | *hsa-miR-7-5p* | *1086.23* | *1.062* | *0.001* | *0.030* | *833.38* | *1562.14* |
|  | hsa-miR-99b-5p | 1141.30 | -1.057 | 0.001 | 0.027 | 1545.39 | 391.51 |
|  |  | | | | | | |
| **Sepsis ARDS vs. COVID-19 ARDS** | **miRNA** | **baseMean** | **log2FC** | **P** | **p_adjusted_** | **Mean Sepsis ARDS** | **Mean COVID-19 ARDS** |
|  | let-7b-3p | 103.09 | -1.172 | <0.001 | 0.006 | 163.17 | 67.89 |
|  | *let-7c-5p* | *310.01* | *-1.214* | *<0.001* | *0.003* | *290.56* | *170.69* |
|  | miR-100-5p | 965.76 | -1.532 | <0.001 | 0.006 | 2009.76 | 407.3 |
|  | miR-1228-5p | 207.86 | 4.284 | <0.001 | 0.002 | 6.48 | 195.89 |
|  | miR-126-3p | 18273.75 | -1.454 | <0.001 | <0.001 | 16101.25 | 7766.67 |
|  | *miR-139-5p* | *555.23* | *-1.505* | *<0.001* | *<0.001* | *719.82* | *282.83* |
|  | *miR-142-3p* | *302.92* | *-2.207* | *<0.001* | *<0.001* | *171.78* | *175.86* |
|  | miR-144-5p | 115.04 | -1.670 | <0.001 | 0.006 | 152.17 | 65.65 |
|  | *miR-146a-5p* | *5987.70* | *1.244* | *<0.001* | *0.005* | *4896.09* | *10221.15* |
|  | *miR-150-5p* | *204.01* | *-2.917* | *<0.001* | *<0.001* | *405.25* | *68.3* |
|  | miR-15b-3p | 96.55 | -1.602 | 0.004 | 0.034 | 142.15 | 71.87 |
|  | miR-182-5p | 961.97 | -1.667 | <0.001 | 0.005 | 1933.38 | 673.12 |
|  | miR-18a-3p | 82.82 | -2.829 | <0.001 | <0.001 | 223.25 | 27.86 |
|  | *miR-191-5p* | *7426.73* | *-1.890* | *<0.001* | *0.006* | *16649.62* | *4818.38* |
|  | miR-192-5p | 3633.91 | -1.397 | 0.002 | 0.026 | 6898.37 | 2521.12 |
|  | miR-194-5p | 1007.82 | -1.423 | 0.001 | 0.012 | 1533.16 | 828.19 |
|  | *miR-20a-5p* | *1027.46* | *1.456* | *<0.001* | *<0.001* | *627.3* | *2805.82* |
|  | *miR-221-3p* | *1458.85* | *1.257* | *0.001* | *0.010* | *1578.03* | *2908.75* |
|  | *miR-27b-3p* | *4690.48* | *-1.189* | *0.001* | *0.011* | *7736.01* | *4850.07* |
|  | miR-30a-3p | 127.69 | -1.178 | 0.001 | 0.016 | 234.26 | 96.02 |
|  | *miR-30c-5p* | *550.32* | *-3.142* | *<0.001* | *<0.001* | *1901.35* | *212.79* |
|  | miR-335-5p | 152.03 | 1.245 | 0.003 | 0.027 | 118.1 | 351.86 |
|  | miR-340-3p | 121.51 | -2.169 | <0.001 | <0.001 | 64.18 | 56.42 |
|  | miR-342-3p | 51.74 | -1.624 | 0.001 | 0.016 | 66.04 | 33.48 |
|  | miR-363-3p | 1478.23 | -1.400 | <0.001 | 0.008 | 1647.88 | 708.54 |
|  | miR-378c | 54.09 | 1.507 | <0.001 | 0.006 | 85.27 | 99.83 |
|  | miR-423-3p | 6416.33 | -1.361 | <0.001 | 0.002 | 10038.41 | 2704.21 |
|  | miR-425-5p | 901.97 | -1.396 | <0.001 | 0.003 | 1822.42 | 506.79 |
|  | miR-4433b-5p | 110.28 | -1.607 | 0.006 | 0.047 | 79.22 | 31.08 |
|  | miR-4732-3p | 173.38 | -1.876 | <0.001 | 0.006 | 338.96 | 39.17 |
|  | miR-484 | 1121.20 | -2.091 | <0.001 | 0.001 | 2319.52 | 614.36 |
|  | *miR-511-5p* | *321.28* | *-1.299* | *0.003* | *0.030* | *674.26* | *330.68* |
|  | miR-542-3p | 78.68 | 1.392 | 0.001 | 0.013 | 71.99 | 216.1 |
|  | miR-654-3p | 137.00 | -1.479 | 0.001 | 0.014 | 91.43 | 38.48 |
|  | miR-92a-3p | 24360.99 | -2.145 | <0.001 | <0.001 | 51367.26 | 6574.07 |
|  | miR-92b-3p | 229.52 | -1.383 | <0.001 | 0.006 | 477.9 | 84.08 |
|  | miR-99a-5p | 17220.77 | -1.598 | <0.001 | 0.007 | 35917.31 | 11720.44 |

miRNAs in italics were chosen for qPCR validation
