# Supplementary material for: Extracellular Vesicle Associated miRNAs Regulate Signaling Pathways Involved in COVID-19 Pneumonia and the Progression to Severe Acute Respiratory Corona Virus-2 Syndrome
Source: Front Immunol. 2021 Dec 9;12:784028. doi: 10.3389/fimmu.2021.784028 (PMC8696174; doi:10.3389/fimmu.2021.784028)
Supplement: Supplementary file 1 [file DataSheet_1.zip › Supplement Meidert et al/e-Table 2 qPCR miRNA.docx]

e-Table 2 shows all RT-qPCR_1_ validation results of the selected significantly differentially regulated miRNAs

| **Comparison** | **miRNA** | **Log2 FC sequencing** | **Log2 FC qPCR** | **p** |
| --- | --- | --- | --- | --- |
| COVID-19 pneumonia  vs.  healthy controls | *miR-1-3p* | *1.2* | *2.11* | *0.432* |
|  | ***miR-193a-5p*** | *1.62* | *2.07* | *0.006* |
|  | ***miR-197-3p*** | *1.71* | *1.66* | *0.021* |
|  | miR-224-5p | -1.57 | 2.52 | 0.879 |
|  | *miR-338-5p* | *1.88* | *2.58* | *0.382* |
|  | *miR-542-3p* | *2.38* | *3.91* | *0.409* |
|  |  |  |  |  |
| COVID-19 ARDS_2_  vs.  COVID-19 pneumonia | let-7e-5p | -1.81 | 0.48 | 0.068 |
|  | ***miR-206*** | *1.58* | *2.73* | *0.025* |
|  | miR-4433b-3p | -1.97 | 2.07 | 0.732 |
|  | miR-4433b-5p | -1.97 | 1.94 | 0.023 |
|  |  |  |  |  |
| COVID-19 ARDS  day 14  vs.  admission on ICU | *miR-146a-5p* | *-1.01* | *-0.08* | *0.923* |
|  | miR-148a-3p | 2.23 | -1.22 | 0.072 |
|  | miR-16-5p | -1.28 | 0.22 | 0.537 |
|  | *miR-17-5p* | *-1.64* | *-0.67* | *0.027* |
|  | *miR-191-5p* | *-1.08* | *-0.39* | *0.045* |
|  | *miR-221-3p* | *-1.32* | *-0.18* | *0.491* |
|  | miR-26b-5p | -1.69 | 1.57 | 0.164 |
|  | miR-98-5p | -1.25 | 0.69 | 0.104 |
|  |  |  |  |  |
| COVID-19 pneumonia  vs.  CAP_3_ | ***let-7g-5p*** | *1.21* | *1.05* | *<0.001* |
|  | miR-15a-5p | 1.34 | -1.15 | 0.558 |
|  | *miR-185-5p* | *1.12* | *1.17* | *0.129* |
|  | *miR-27a-3p* | *-1.04* | *-0.17* | *0.051* |
|  | *miR-7-5p* | *1.06* | *-0.53* | *0.006* |
|  |  |  |  |  |
| COVID-19 ARDS  vs.  Sepsis | let-7c-5p | -1.21 | 2.33 | 0.001 |
|  | *miR-139-5p* | *-1.50* | *-1.16* | *0.227* |
|  | miR-142-3p | -2.21 | 0.13 | 0.234 |
|  | *miR-150-5p* | *-2.92* | *-3.07* | *0.788* |
|  | ***miR-20a-5p*** | *1.46* | *1.08* | *<0.001* |
|  | *miR-27b-3p* | *-1.19* | *-1.92* | *0.079* |
|  | *miR-30c-5p* | *-3.14* | *-1.04* | *0.843* |
|  | miR-511-5p | -1.30 | 0.00 | <0.001 |

miRNAs in italics show the same regulatory direction in NGS and qPCR

miRNA in bold meet the sequencing cutoff values of log2FC ≥ 1 or log2FC ≤ -1 and p ≤ 0.05

_1_qPCR = polymerase chain reaction

_2_ARDS = acute respiratory distress syndrome

_3_CAP = Community acquired pneumonia of bacterial origin
