# Supplementary material for: Extracellular Vesicle Associated miRNAs Regulate Signaling Pathways Involved in COVID-19 Pneumonia and the Progression to Severe Acute Respiratory Corona Virus-2 Syndrome
Source: Front Immunol. 2021 Dec 9;12:784028. doi: 10.3389/fimmu.2021.784028 (PMC8696174; doi:10.3389/fimmu.2021.784028)
Supplement: Supplementary file 1 [file DataSheet_1.zip › Supplement Meidert et al/e-Table 3 qPCR mRNA.docx]

e-Table 3 shows all RT-qPCR validation results of the selected in-silico identified *target mRNAs* of differentially expressed miRNAs

|  | **Gen** | **qPCR** | | | |
| --- | --- | --- | --- | --- | --- |
|  |  | **log2FC** | **p** | **ΔCq_Pneumonia_** | **ΔCq_Controls_** |
| COVID-19 pneumonia  vs.  healthy controls | **IL6** | -1.231 | 0.024 | 12.04 | 10.80 |
|  | TNF | -0.415 | 0.105 | 5.31 | 4.89 |
|  | CD40 | -0.242 | 0.450 | 6.29 | 6.05 |
|  | CXCR4 | -0.245 | 0.204 | 1.21 | 0.97 |
|  | IFNB1 | -0.710 | 0.089 | 13.09 | 12.38 |
|  | IL1R1 | 0.017 | 0.943 | 7.40 | 7.41 |
|  | **PIK3R1** | -1.081 | 0.001 | 5.52 | 4.44 |
|  | STAT1 | 0.683 | 0.059 | 1.49 | 2.18 |
|  | TLR7 | 0.072 | 0.845 | 5.86 | 5.93 |
|  | **CCR3** | -1.587 | 0.009 | 5.86 | 4.27 |
|  | CXCL8/IL8 | -1.329 | <0.001 | 7.71 | 6.38 |
|  | **JAK1** | -1.111 | 0.001 | 2.61 | 1.50 |
|  | **PPARG** | 3.403* | <0.001 | 8.95 | 12.36 |
|  | EGFR |  |  | n.a._1_ | n.a. |
|  | NOS2 |  |  | n.a. | n.a. |
|  | F2 |  |  | n.a. | n.a. |
|  | IL36A |  |  | n.a. | n.a. |
|  |  |  |  | **ΔCq_ARDS_** | **ΔCq_Pneumonia_** |
| COVID-19 ARDS_2_  vs.  COVID-19 pneumonia | HIF1A | 0.252 | 0.174 | 1.83 | 2.08 |
|  | PTGS2 | -0.244 | 0.388 | 5.15 | 4.91 |
|  | TGFB2 | -0.006 | 0.992 | 11.31 | 11.31 |
|  | **CXCL8/IL8** | 0.898 | 0.040 | 5.86 | 6.76 |
|  | PDCD1 | -0.334 | 0.411 | 10.93 | 10.60 |
|  | IL17F |  |  | n.a. | n.a. |

mRNAs in bold meet the cutoff of log2FC ≥ 1 or log2FC ≤ -1 and p ≤ 0.05

_1_n.a.= not expressed

_2_ARDS = acute respiratory distress syndrome

*opposite direction of expression as predicted *in-silico*
